# Sperm DNA Fragmentation Impairs Early Embryo Development but Is Not Predictive of Pregnancy Outcomes: Insights from 870 ICSI Cycles

**DOI:** 10.3390/ijms26167923

**Published:** 2025-08-16

**Authors:** Tomasz Machałowski, Julita Machałowska, Kamil Gill, Maciej Ziętek, Małgorzata Piasecka, Grzegorz Mrugacz, Przemysław Ciepiela

**Affiliations:** 1 Department of Perinatology, Obstetrics and Gynecology, Pomeranian Medical University in Szczecin, 70-204 Szczecin, Poland; tomasz.machalowski@pum.edu.pl (T.M.); maciej.zietek@pum.edu.pl (M.Z.); 2 Bocian Fertility Clinic, Gynecology and Obstetrics, Brama Portowa 1, 70-225 Szczecin, Poland; jmachalowska@klinikabocian.pl (J.M.); gmrugacz@klinikabocian.pl (G.M.); 3 Department of Histology and Developmental Biology, Faculty of Health Sciences, Pomeranian Medical University, 71-210 Szczecin, Poland; kamil.gill@pum.edu.pl (K.G.); mpiasecka@ipartner.com.pl (M.P.)

**Keywords:** sperm DNA fragmentation, intracytoplasmic sperm injection (ICSI), embryo development, clinical pregnancy, male infertility, fertilization rate, blastocyst formation, embryo quality, reproductive outcomes

## Abstract

Sperm DNA fragmentation (SDF) is increasingly regarded as a biomarker of male infertility, but its predictive value for intracytoplasmic sperm injection (ICSI) outcomes remains controversial. This retrospective cohort study analyzed 870 fresh single-blastocyst ICSI cycles performed between January 2023 and December 2024. SDF was measured using the Sperm Chromatin Dispersion test and patients were categorized into low (SDF ≤ 20%, *n* = 664) and high (SDF > 20%, *n* = 206) groups. Higher SDF was significantly associated with reduced semen quality, lower fertilization rates, and poorer blastocyst development. In multivariable analysis, each 1% increase in SDF reduced the odds of achieving a fertilization rate > 80% by 1.6% (OR = 0.984, 95% CI: 0.971–0.997, *p* = 0.015) and decreased the chance of obtaining top-quality blastocysts on day 5 by 2.5% (OR = 0.975, 95% CI: 0.958–0.992, *p* = 0.004). A trend toward impaired day-3 embryo quality was observed (OR = 0.983, *p* = 0.068). No significant association was found with clinical pregnancy (OR = 0.989, *p* = 0.155), while the relationship with miscarriage was borderline (OR = 0.961, *p* = 0.053). These findings suggest that elevated SDF adversely impacts early embryological outcomes in ICSI, supporting its use as a prognostic tool during ART counseling.

## 1. Introduction

Infertility is an increasing global health issue, affecting about 15–20% of couples of reproductive age. As access to and demand for assisted reproductive technologies (ARTs) grow, so does the need to identify reliable biomarkers of reproductive success [1,2,3]. The male factor accounts for 20% to 70% of infertility cases, emphasizing the importance of thorough evaluation of sperm quality. While standard semen analysis measures concentration, motility, and morphology, these parameters do not fully indicate sperm’s functional ability. A more direct indicator of sperm genomic health, sperm DNA fragmentation (SDF), has become an important biomarker for male fertility potential [1,4,5].

SDF has become a crucial factor in assessing male reproductive potential, with increasing evidence linking high SDF levels to lower fertilization rates, impaired embryo development, and, in some cases, poorer pregnancy outcomes in ART cycles. Several factors contribute to elevated SDF, including oxidative stress, defective spermatogenesis, and paternal aging. Advanced paternal age (APA) is particularly associated with higher baseline SDF, reduced tolerance to cryopreservation stress, and mitochondrial dysfunction [6]. Technological advances in sperm selection now use both microfluidic devices, which decrease SDF while selecting sperm with better motility and morphology, and physiological methods like exposing sperm to the cumulus cell secretome during swim-up, which enhances capacitation, acrosome reaction, mitochondrial activity, and tyrosine phosphorylation, while reducing DNA damage [7,8,9]. These findings emphasize the complex nature of SDF and highlight the importance of considering both individual patient risk factors and laboratory techniques when analyzing SDF results and refining ART strategies.

SDF refers to single- and double-stranded DNA breaks in sperm chromatin and can result from oxidative stress, defective spermatogenesis, abortive apoptosis, or environmental and lifestyle factors [4,10]. Multiple assays are available for its detection, including the sperm chromatin dispersion (SCD) test, sperm chromatin structure assay (SCSA), TUNEL, and the Comet assay [11,12]. In the context of ART, particularly intracytoplasmic sperm injection (ICSI), elevated SDF has been linked to reduced fertilization rates, impaired embryo development, and, in some studies, lower pregnancy and live birth rates [13,14,15].

Despite increasing recognition of its importance, the clinical utility of SDF remains a topic of debate. Existing studies have been constrained by small sample sizes, variable cut-off thresholds, and methodological heterogeneity [16,17]. Many reports include fewer than 200–300 subjects, limiting statistical power and generalizability. Furthermore, a consensus on clinically significant SDF thresholds is lacking, with proposed values ranging from 15% to 30%, depending on the assay and study population [4,12,18,19,20].

There is thus a pressing need for extensive, standardized studies to validate the prognostic value of SDF and determine evidence-based thresholds. Our study addresses this gap by evaluating the association between SDF levels and ICSI outcomes in a large, homogeneous cohort of nearly 900 couples, using a consistent diagnostic and clinical protocol. Specifically, we aimed to identify whether SDF thresholds could serve as reliable predictors of fertilization, embryo development, and clinical pregnancy outcomes. To our knowledge, this is one of the most extensive investigations to date systematically examining the impact of sperm DNA integrity on ICSI success and its clinical implications.

## 2. Results

### 2.1. Baseline Characteristics and Clinical Outcomes

This study included 870 couples undergoing ICSI cycles. Baseline demographics and clinical outcomes are shown in Table 1. The mean female age was 36.1 ± 4.7 years, and the mean male age was 38.1 ± 5.9 years. Data on infertility duration were available for 429 couples, with an average duration of 3.8 ± 2.4 years. Semen Analysis parameters were recorded for all 870 cycles, showing a mean sperm concentration of 52.3 ± 56.4 × 10^6^/mL, fast progressive motility of 11.9 ± 9.7%, and normal sperm morphology of 2.6 ± 1.1%. The mean sperm DNA fragmentation was 16.2 ± 10.4%. The fertilization rate per injected oocyte was 78.3 ± 24.4%, with a median of 83.3%. The blastocyst development rate per fertilized metaphase II oocyte was 57.4% (median 60%), while good-quality blastocysts accounted for 26.3 ± 25.6% of all embryos. The implantation rate, calculated per embryo transfer for all 870 cycles, was 49.1%. Clinical pregnancy data were available for 659 transfers, with a clinical pregnancy rate of 57.2%. Clinical pregnancy was confirmed by ultrasound detection of a fetal heartbeat. Among 377 clinical pregnancies, 49 resulted in miscarriage, defined as spontaneous pregnancy loss before 20 weeks of gestation, yielding a miscarriage rate of 13.0%. Live birth data were not available for all cases and are therefore not reported here. The difference between implantation and clinical pregnancy rates arises from the different denominators: the implantation rate was calculated for all embryo transfers, while the clinical pregnancy rate was based only on cases with a confirmed fetal heartbeat via ultrasound.

### 2.2. Comparison of Mean SDF Values Across Fertilization, Embryo Quality, and Pregnancy Outcomes

**Figure 1:**
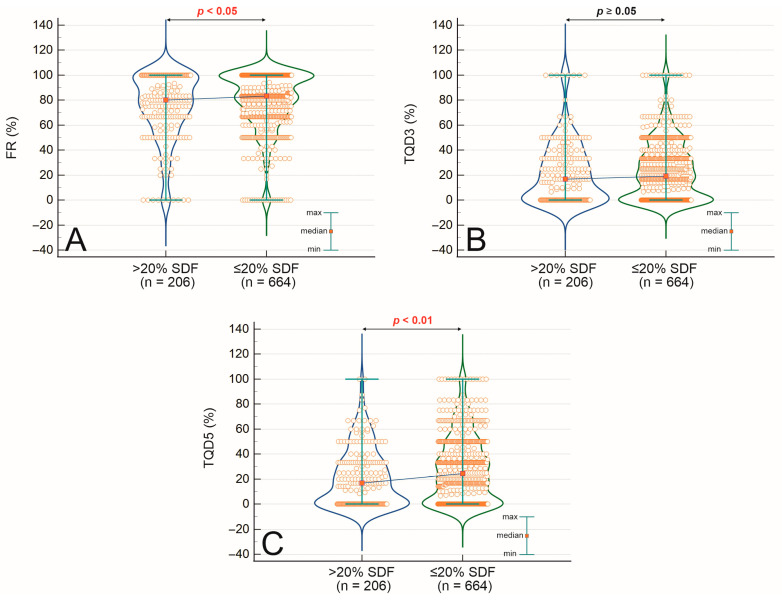
Comparison of sperm DNA fragmentation (SDF) between groups dependent on fertilization rate (FR) (**A**), top-quality embryos on day 3 (TQD3) (**B**), and top-quality embryos on day 5 (TQD5) (**C**). Statistical significance was noted in the Mann–Whitney U test when *p* < 0.05.

Table 2 summarizes the mean sperm DNA fragmentation values across groups divided by fertilization rate, embryo quality on days 3 and 5, and clinical outcomes of ICSI cycles. When grouped by fertilization rate, patients with FR < 80% (*n* = 384) had a mean SDF of 17.04 ± 10.50, compared to 15.47 ± 10.24 in patients with FR ≥ 80% (*n* = 486), showing a statistically significant difference (*p* = 0.009). In cycles with a lower proportion of top-quality blastocysts on day 5 (TQD5 < 50%, *n* = 751), the mean SDF was 16.41 ± 10.56, while in cycles with TQD5 ≥ 50% (*n* = 119), it was slightly lower at 14.60 ± 9.05, showing a trend toward significance (*p* = 0.057). A similar pattern emerged for top-quality embryos on day 3 (TQD3), with mean SDF values of 16.26 ± 10.30 (TQD3 < 50%, *n* = 799) and 15.13 ± 11.20 (TQD3 ≥ 50%, *n* = 71), respectively (*p* = 0.184). Regarding reproductive outcomes, the mean SDF in patients without confirmed pregnancy (*n* = 303) was 16.47 ± 10.33, whereas in those with pregnancy (*n* = 406), it was 15.40 ± 9.56 (*p* = 0.210). Among patients with a clinical pregnancy (*n* = 377), the mean SDF was 16.39 ± 10.39, compared to 13.33 ± 7.29 in those who experienced a miscarriage (*n* = 49), with a significant difference (*p* = 0.037). These distributions are illustrated in Figure 1A–D, which compare SDF values across groups based on fertilization rate (Figure 1A), top-quality embryos on day 3 (Figure 1B), top-quality blastocysts on day 5 (Figure 1C), and pregnancy outcome (Figure 1D). Statistical comparisons in these figures were made using the Mann–Whitney U test, with *p* < 0.05 considered significant.

### 2.3. Associations and Correlations Between Sperm DNA Fragmentation, Semen Quality, Embryo Development, and Clinical Outcomes

**Figure 2:**
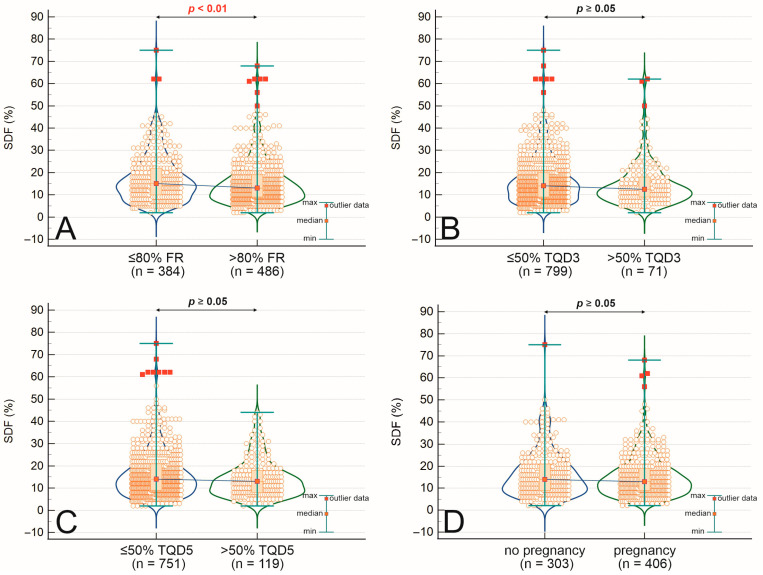
Comparison of fertilization rate (FR) (**A**), top-quality embryos on day 3 (TQD3) (**B**), and top-quality embryos on day 5 (TQD5) (**C**) between groups dependent on sperm DNA fragmentation (SDF) and pregnancy outcomes (**D**). Statistical significance was noted in the Mann–Whitney U test when *p* < 0.05.

Table 3 presents a comprehensive comparison of semen parameters, embryological development metrics, and clinical outcomes between patients with high sperm DNA fragmentation (SDF > 20%) and those with low SDF (≤20%). Men in the high SDF group were significantly older than those in the low SDF group (39.8 ± 6.6 vs. 37.6 ± 5.5 years, *p* < 0.001), while no difference in body mass index (BMI) was observed. High SDF was associated with significantly lower sperm concentration (46.7 ± 62.6 vs. 53.9 ± 54.5 × 10^6^/mL, *p* < 0.001), fast progressive motility (8.9 ± 9.1% vs. 12.7 ± 9.7%, *p* < 0.001), and slow progressive motility (14.9 ± 10.9% vs. 21.5 ± 11.7%, *p* < 0.001), as well as a higher teratozoospermia index (TZI) (2.84 ± 1.73 vs. 2.56 ± 1.68, *p* = 0.020). Although the percentage of morphologically normal sperm did not differ significantly (2.00 ± 1.92% vs. 2.00 ± 1.88%, *p* = 0.083), SDF was negatively correlated with morphology (r = −0.137, *p* < 0.001). Embryologically, higher SDF was associated with significantly lower fertilization rates (74.5 ± 26.7% vs. 79.4 ± 23.6%, *p* = 0.019) and top-quality blastocyst rates on day 5 (21.3 ± 23.1% vs. 27.8 ± 26.1%, *p* < 0.001), while the difference in top-quality embryo rate on day 3 did not reach statistical significance (16.7 ± 25.5% vs. 19.1 ± 23.4%, *p* = 0.187). Clinical pregnancy rates showed a trend toward reduction in the high SDF group (51.9% vs. 58.9%), although this difference was not statistically significant (*p* = 0.113). These interrelations are further visualized in Figure 2A–C. Correlation analyses further revealed substantial associations between SDF and multiple sperm quality parameters, including negative correlations with motility, morphology, and fertilization outcomes, as well as a positive correlation with male age and the count of immotile sperm.

### 2.4. Logistic Regression Analysis

Multivariable logistic regression analysis revealed that higher sperm DNA fragmentation was significantly associated with lower chances of achieving key reproductive outcomes in ICSI cycles (Table 4). Notably, SDF had a negative correlation with fertilization efficiency: each 1% increase in SDF reduced the odds of reaching a fertilization rate over 80% by 1.6% (OR = 0.984, 95% CI: 0.971–0.997, *p* = 0.015). Similarly, the likelihood of obtaining top-quality blastocysts on day 5 decreased significantly with higher SDF (OR = 0.975, 95% CI: 0.958–0.992, *p* = 0.004), amounting to a 22.5% reduction in odds per 10% increase in SDF. A trend towards a negative association was also seen for top-quality embryo formation on day 3 (OR = 0.983, *p* = 0.068), though it was not statistically significant. Additionally, while the risk of miscarriage appeared higher with increasing SDF (OR = 0.961, *p* = 0.053), the result was borderline significant and should be interpreted with caution. Importantly, no significant link was found between SDF levels and clinical pregnancy rates (OR = 0.989, *p* = 0.155), indicating that once implantation happens, moderate SDF levels may have limited impact on establishing pregnancy. These findings emphasize the adverse effect of elevated SDF mainly on early reproductive outcomes—fertilization and embryo development—while the effects on post-implantation results seem less clear or more variable.

## 3. Discussion

This extensive, single-center retrospective study confirms a significant link between elevated sperm DNA fragmentation and decreased semen quality, lower fertilization success, and poorer embryo development in ICSI cycles. However, we did not find a statistically significant direct link between SDF levels and clinical pregnancy rates. These findings support the growing consensus that while SDF is an important indicator of gamete and embryo competence, it alone may not be enough to predict pregnancy outcomes, which are inherently influenced by multiple factors and both partners.

### 3.1. SDF and Sperm Quality

Our data reaffirm well-established links between high SDF levels and abnormal semen parameters, including lower concentration, motility, and morphology. This relationship, consistently shown in previous studies [21,22,23,24], highlights the biological plausibility of SDF as a marker of impaired spermatogenesis and oxidative stress. Green et al. and Zhang et al. have observed that SDF may worsen before standard semen parameters fall below diagnostic thresholds, suggesting its usefulness as a complementary biomarker in male infertility evaluations [25,26].

### 3.2. SDF and Fertilization Efficiency

Although ICSI was created to overcome limitations from poor sperm quality, our findings confirm it does not entirely eliminate the negative effect of sperm DNA damage on fertilization outcomes. Consistent with previous meta-analyses and observational studies, we saw significantly lower fertilization rates in men with high SDF levels [16,26,27,28,29]. Fragmented DNA may hinder chromatin remodeling and oocyte activation, resulting in failed or delayed zygotic development even in the highly controlled setting of ART [30,31]. Ribas-Maynou et al. suggested that SDF may interfere with early events such as paternal genome reprogramming and zygotic gene activation, supporting the biological plausibility of our findings [15,32].

### 3.3. SDF and Embryo Development

The negative effects of SDF extended beyond fertilization, as shown by significantly lower rates of high-quality embryos and blastocysts in the high SDF group. These results support earlier reports by Sakkas and Alvarez, Sedó et al., and Ribas-Maynou et al. which suggest that DNA fragmentation IVF (in vitro fertilization) harms embryo viability [15,31,33]. Ni et al. saw similar outcomes in ICSI, but not in IVF cycles, highlighting that sperm chromatin integrity is particularly important when natural selection barriers are skipped. Mechanistically, damaged sperm DNA may cause defective gene expression, disrupted cleavage processes, and poor epigenetic remodeling in early embryos [34,35,36].

### 3.4. SDF and Clinical Pregnancy: A Multifactorial Equation

Despite these clear effects on early ART outcomes, we did not find a statistically significant link between SDF and clinical pregnancy rates. This result aligns with several large studies and meta-analyses [15,17,29] and suggests that ICSI might reduce some of the downstream effects of SDF. Additionally, the average SDF difference between pregnant and non-pregnant groups (~1%) may not be clinically significant. These findings support the idea that achieving a clinical pregnancy is influenced by a complex interaction of factors, including endometrial receptivity, immune status, female age, hormonal environment, and embryo–endometrial communication. Importantly, oocytes have a limited but meaningful ability to repair fragmented paternal DNA after fertilization, which could offset mild to moderate sperm DNA damage [30,37,38]. However, high SDF may still indirectly lower pregnancy rates by affecting embryo quality and transfer success.

Our findings align with previous large-scale evidence showing that elevated SDF impairs early embryological competence, particularly by reducing fertilization rates and compromising blastocyst quality, although its connection to clinical pregnancy remains inconsistent. These results support the idea that SDF operates within a multifactorial framework, where male factors interact with female reproductive parameters, laboratory conditions, and embryo transfer protocols to influence ART outcomes. Notably, recent research by Luongo et al. demonstrated that adding the cumulus cell secretome during swim-up selection significantly improves sperm capacitation, acrosome reaction, mitochondrial activity, and tyrosine phosphorylation, while decreasing DNA fragmentation, providing a physiologically based approach for selecting functionally competent spermatozoa. In addition, Iranzo et al. found that microfluidic sperm selection devices effectively reduce SDF and enhance motility and morphology compared to traditional methods, with the potential to improve ART outcomes in certain patient groups. Moreover, Casasús et al. highlighted the negative impact of APA on sperm genomic stability, including increased baseline SDF, reduced resilience to cryopreservation, and more mitochondrial damage. Collectively, these studies emphasize that combining SDF assessment with targeted sperm selection techniques—whether microfluidic, cumulus cell secretome-enhanced, or both—may be especially useful in high-risk groups, such as older men or those with elevated SDF. Future high-quality, prospective trials should investigate how these advanced selection methods work together to improve live birth rates, which remain the most vital measure of ART success.

Emerging microfluidic sperm selection technologies offer promising opportunities to improve ART outcomes in cases with high SDF. Several studies show that microfluidic methods outperform traditional approaches. For instance, Ruiz-Díaz et al. reported a 13.3% reduction in DNA fragmentation using thermotaxis-based microfluidics in bull sperm [39], while Dehghanpour et al. demonstrated improved human sperm motility and reduced DNA fragmentation with a centrifuge-free microfluidic sorting technique [40]. Microfluidic devices also utilize rheotaxis to effectively separate motile sperm from seminal plasma components that can impede fertilization [40,41,42]. Biomimetic platforms, which better replicate the environment of the female reproductive tract, have resulted in lower DNA fragmentation rates compared to swim-up methods [7]. Conversely, density gradient centrifugation can sometimes increase DNA fragmentation [43,44]. Additional improvements through apoptotic marker selection have also been shown to enhance sperm quality and reproductive success [45,46]. A recent meta-analysis of 39 studies confirmed that microfluidics significantly decrease DNA fragmentation and improve motility, morphology, fertilization, implantation, clinical pregnancy, and live birth rates compared to traditional methods [7]. However, the evidence remains insufficient to recommend routine clinical use due to gaps in cost-effectiveness and long-term outcomes. Future research should focus on optimizing these technologies and evaluating their clinical utility, particularly in patients with high sperm DNA fragmentation.

### 3.5. Practical Implications for ART

Our findings offer several important implications for reproductive medicine:

-Threshold Re-evaluation: The widely cited SDF threshold of 30% seems too cautious. Clinically meaningful impairments were seen at lower levels (15–20%), supporting recent suggestions to update cut-off values [4,18,20,47].

-Personalized Treatment Strategies: SDF testing can help customize ART protocols, especially in cases of unexplained infertility or repeated ART failures. Additional strategies—such as antioxidant supplements, testicular sperm retrieval, and advanced sperm selection methods (e.g., MACS—Magnetic Activated Cell Sorting, PICSI—Physiological Intracytoplasmic Sperm Injection)—may lower SDF levels and enhance success rates [2,22,34].

-Couple-Centered Assessment: SDF should not be assessed in isolation. Female reproductive potential—particularly age, ovarian reserve, and endometrial health—affects the clinical significance of sperm DNA damage. Incorporating SDF into a comprehensive, couple-based diagnostic approach may improve reproductive counseling and success prediction [48,49].

### 3.6. Strengths, Limitations, and Future Directions

Our study is among the largest single-center analyses so far examining the link between SDF and ICSI outcomes. Using a uniform cohort, standardized stimulation and lab protocols, and consistent SDF assessment (SCD test) improves internal validity and reduces inter-laboratory variability.

However, some limitations need to be noted. The retrospective design may introduce selection and reporting biases. Although practical and widely used, the SCD assay might not detect all aspects of DNA damage. We also lacked mechanistic data—such as markers of oxidative stress or epigenetic profiling—that could clarify pathways connecting SDF to embryo development. In addition, not all outcomes, such as miscarriage or live birth, were included in this dataset. Another limitation of the current study is the lack of detailed CASA-derived kinematic parameters, including average path velocity (VAP), curvilinear velocity (VCL), straight-line velocity (VSL), and linearity (LIN). While such measures can offer deeper mechanistic insights into sperm motility characteristics and their connection with DNA integrity, our analysis was limited to WHO 6th Edition motility categories (fast progressive, slow progressive, non-progressive, and immotile sperm). This reflects standard clinical reporting at our center during the study period. Nonetheless, the large sample size and consistent motility classification across all 870 cycles help ensure internal validity. Future prospective studies at our institution will incorporate comprehensive CASA profiling to understand motility–SDF relationships better.

Future research should focus on standardizing sperm DNA fragmentation testing protocols across laboratories to enhance comparability and clinical usefulness. Simultaneously, multi-omics approaches are necessary to uncover the molecular mechanisms behind SDF-related embryopathy, deepening our understanding of how paternal DNA damage affects early embryonic development. Importantly, clinical studies must evaluate whether interventions aimed at reducing SDF, such as antioxidant treatments or advanced sperm selection techniques, can lead to improved live birth rates. Randomized controlled trials are urgently needed to determine their effectiveness. Additionally, emerging evidence indicates that the oocyte’s ability to repair DNA may influence the clinical outcomes of high SDF, especially in younger women [50]. Oocytes from women under 30 likely have a better capacity to repair paternal DNA damage, allowing for successful pregnancies even when sperm DNA fragmentation exceeds 20–30%. Age-specific research focusing on this interaction could provide valuable insights, enabling more personalized counseling and tailored ART strategies considering both male and female reproductive factors.

## 4. Materials and Methods

### 4.1. Study Design and Participants

This retrospective study analyzed clinical data from 3972 ICSI cycles conducted at the Infertility Treatment Clinic BOCIAN in Szczecin and Warsaw, Poland, between January 2023 and December 2024. Among these, 870 cycles with significant sperm DNA fragmentation were included in the analysis. Baseline characteristics and clinical outcomes are summarized in Table 1. The median ages of females and males were 35.9 and 37.3 years, respectively. The mean duration of infertility was 3.82 ± 2.39 years (median 3.12, *n* = 429). The study protocol was approved by the Ethics Committee of the Pomeranian Medical University in Szczecin (KB.006.052.2025; 28 February 2025), and all participants provided written informed consent. Only ICSI cycles using partner’s sperm with fresh Day 5 embryo transfer were included. Cycles involving cryopreservation or Day 3 embryo transfer were excluded.

### 4.2. Assessment of Sperm DNA Fragmentation

**Figure 3:**
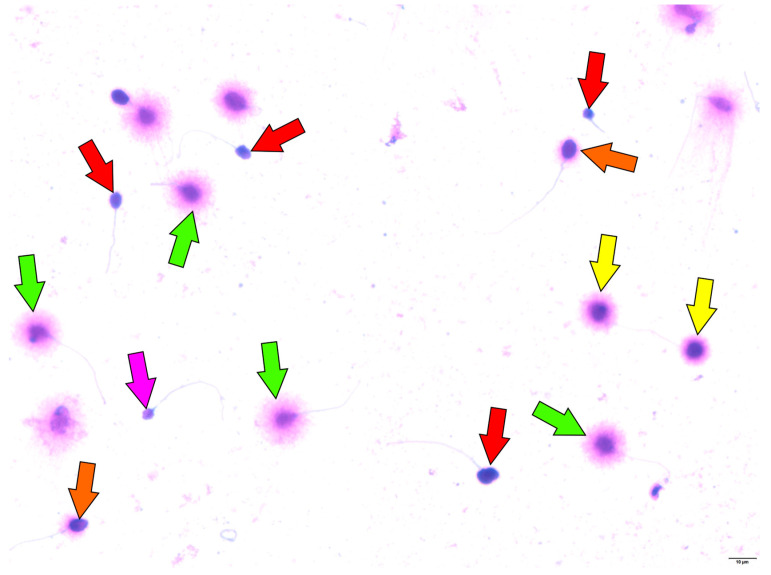
Representative light microscopy images of sperm chromatin dispersion (SCD) test results. Spermatozoa with large halos (green arrows) or medium halos (yellow arrows) have intact nuclear DNA. In contrast, those with small halos (orange arrow), no halos (red arrows), or no halos with degraded chromatin (violet arrow) show fragmented nuclear DNA. Image source: original micrographs by Kamil Gill.

Sperm motility and morphology were evaluated following the *World Health Organization Laboratory Manual for the Examination and Processing of Human Semen*, 6th Edition (2021) [51]. Motility was expressed as the percentage of fast progressive, slow progressive, non-progressive, and immotile sperm, assessed using computer-assisted semen analysis (CASA). Detailed kinematic parameters such as VAP, VCL, VSL, and LIN were not recorded in the clinical database during the study, which is acknowledged as a limitation in the discussion. Morphology was assessed using strict WHO criteria by trained embryologists with established inter-observer reproducibility. Sperm DNA fragmentation was assessed using the Sperm Chromatin Dispersion (SCD) test with the Halosperm G2 kit (Halotech DNA, Madrid, Spain) following the manufacturer’s instructions [11,52]. Briefly, sperm samples were diluted to ≤20 million/mL and mixed with melted agarose. A 10 µL aliquot was applied to super-coated slides, then subjected to denaturation, lysis, dehydration, and staining with eosin and thiazine. The slides were examined under bright-field microscopy at 1000× magnification (CX31, Olympus, Tokyo, Japan), with at least 300 spermatozoa counted per sample. Spermatozoa without DNA fragmentation formed halos of dispersed DNA loops classified as large (halo width ≥ sperm head diameter) or medium (halo width > 1/3 sperm head diameter) (Figure 3: green and yellow arrows). Sperm with fragmented DNA showed small halos (≤1/3 sperm head diameter), no halos, or degraded morphology (Figure 3, orange, red, violet arrows) [11,52]. The SDF index was calculated as the percentage of spermatozoa with fragmented DNA relative to the total counted.

### 4.3. Controlled Ovarian Stimulation and ICSI Procedure

Ovarian stimulation was performed using a combination of FSH and LH, with pituitary suppression through one of three protocols: microdose flare, fixed antagonist, or mid-luteal long agonist. Final oocyte maturation was triggered with hCG once at least two follicles reached ≥18 mm in diameter. Cumulus cells were stripped from retrieved oocytes approximately 2 h after retrieval. ICSI was performed at least 1 h after cumulus removal, inseminating only metaphase II (MII) oocytes with the partner’s sperm. Immature oocytes were discarded. Fertilization was assessed 16–18 h post-insemination by the presence of two pronuclei and two polar bodies [24,53].

### 4.4. Embryo Culture and Transfer

Zygotes were cultured in cleavage media (G-TL™, Vitrolife, Sweden) and monitored using the EmbryoScope + time-lapse system (Vitrolife, Sweden) on Days 2, 3, 4, and 5 [26]. Single-embryo transfer was performed on Day 5 using an Emtrac catheter (Gynetics, Belgium) under ultrasound guidance. Embryos were graded on Day 2 (4-cell stage) and Day 3 (8-cell stage) for fragmentation (<20%) and morphology (Grade 1 or 2, defined as good quality) [25]. Blastocyst quality was assessed on Day 5 according to Gardner’s criteria [28], with top-quality blastocysts defined as 4AA, 5AA, 4AB, 4BA, 5AB, or 5BA [15,54]. Good-quality surplus blastocysts were cryopreserved. Fertilization rate was calculated as the proportion of 2PN oocytes among MII oocytes. Blastocyst development rate was the ratio of blastocysts to fertilized oocytes. Clinical pregnancy was defined by the presence of an intrauterine gestational sac with fetal heartbeat confirmed by ultrasound. The implantation rate was calculated as the number of gestational sacs per embryos transferred. Miscarriage was defined as pregnancy loss prior to 20 weeks of gestation after confirmation of intrauterine pregnancy [27].

### 4.5. Statistical Analysis

The normality of data distribution was evaluated using the Shapiro–Wilk test. Because distributions were non-normal, nonparametric tests were utilized. Continuous variables were compared with the Mann–Whitney U test, while categorical variables were analyzed using the Chi-square test. Logistic regression was employed for dichotomous outcomes, and linear regression was used for continuous dependent variables. Correlations were examined using Spearman’s rank correlation coefficient. Statistical significance was set at *p* < 0.05. Analyses were conducted with Statistica version 13.3 (StatSoft, Kraków, Poland) and MedCalc version 22.009 (MedCalc Software, Ostend, Belgium).

## 5. Conclusions

Our study highlights the clinical significance of sperm DNA fragmentation as an important indicator of male reproductive health. Elevated SDF levels were strongly associated with poorer semen quality, lower fertilization rates, and less optimal embryonic development in fresh ICSI cycles. However, we found no statistically significant impact of SDF on clinical pregnancy or miscarriage rates, underscoring the complex and multifaceted nature of implantation and pregnancy maintenance, likely influenced more by female reproductive factors. Importantly, our results suggest the need to reevaluate existing SDF thresholds. Notable differences in fertilization and embryo quality emerged at lower fragmentation levels (15–20%), consistent with recent studies [4,19,20,47,55,56,57]. Refining this threshold could enhance diagnostic precision and patient counseling in ART practice. Routine SDF testing may offer valuable insights as part of a comprehensive, couple-centered fertility assessment—especially in cases of unexplained infertility or repeated ART failures. Future prospective and longitudinal studies are needed to track SDF fluctuations over time and examine how factors such as age, lifestyle, environmental exposures, and treatments impact sperm DNA integrity. Gaining this knowledge will be essential for advancing personalized reproductive medicine and improving outcomes for couples undergoing assisted reproduction.

## Figures and Tables

**Table 1:** Baseline characteristics and clinical outcomes of the study population (*n* = 870).

Parameter	No. of Cycles	Mean ± SD	Rate [%]
Female age [years]	870	36.10 ± 4.72	—
Male age [years]	870	38.12 ± 5.88	—
Duration of infertility [years]	429 *	3.82 ± 2.39	—
Sperm concentration [×10^6^/mL]	870	52.30 ± 56.44	—
Fast progressive motility [%]	870	11.85 ± 9.71	—
Normal sperm morphology [%]	870	2.56 ± 1.12	—
Sperm DNA fragmentation (SDF) [%]	870	16.17 ± 10.38	—
Fertilization rate [% per oocyte injected]	870	78.25 ± 24.42	83.33 (median)
Blastocyst development rate [% per fertilized MII oocyte]	870	—	57.41 (mean); 60 (median)
Good-quality blastocyst [%]	870	26.28 ± 25.57	—
Implantation rate [% per transfer]	870	—	49.06
Clinical pregnancy rate [% per transfer]	377/659	—	57.21
Miscarriage rate [% per clinical pregnancy]	49/377	—	12.99

Footnotes: Data were not available for all couples; parameters marked with an asterisk (*) represent a subset of the total study population. The implantation rate was calculated per embryo transfer and defined as the presence of a gestational sac visible on ultrasound (USG). The clinical pregnancy rate was calculated for 659 transfers with known fetal heartbeat outcomes detected by ultrasound. Clinical pregnancy was defined as a fetal heartbeat detected on ultrasound. Live birth was defined as the delivery of a live infant. Miscarriage was defined as spontaneous pregnancy loss before the 20th week of gestation. Abbreviations: SD, standard deviation; SDF, sperm DNA fragmentation; MII, metaphase II oocyte.

**Table 2:** Sperm DNA fragmentation in relation to Fertilization rate, embryo quality, and reproductive outcomes in ICSI cycles.

Comparison Group	Group A	Group B	SDF [%] (Mean ± SD)	*p*-Value *
Fertilization Rate (FR)	FR < 80% (*n* = 384)	FR ≥ 80% (*n* = 486)	17.04 ± 10.50 vs. 15.47 ± 10.24	0.009
Top-quality embryos day 3 (TQ3)	TQD3 < 50% (*n* = 799)	TQD3 ≥ 50% (*n* = 71)	16.26 ± 10.30 vs. 15.13 ± 11.20	0.184
Top-quality blastocysts (TQ5)	TQD5 < 50% (*n* = 751)	TQD5 ≥ 50% (*n* = 119)	16.41 ± 10.56 vs. 14.60 ± 9.05	0.056
Pregnancy Outcome	No pregnancy (*n* = 303)	Pregnancy (*n* = 406)	16.47 ± 10.33 vs. 15.40 ± 9.56	0.210
Pregnancy Result	Clinical Pregnancy (*n* = 377)	Miscarriage (*n* = 49)	16.39 ± 10.39 vs. 13.33 ± 7.29	0.037

*p*-value * based on the Mann–Whitney U test; *p* < 0.05 was considered statistically significant. Abbreviations: SDF—sperm DNA fragmentation; FR—fertilization rate; TQ3—top-quality embryos on day 3 of culture; TQ5—top-quality blastocysts on day 5 of culture; TQD3, TQD5—proportion of top-quality embryos/blastocysts; SD—standard deviation. Pregnancy = clinically confirmed gestation on ultrasound. Note: Values comparing “Pregnancy” vs. “No pregnancy” refer to historical cycle records regarding the occurrence of any confirmed pregnancy. In contrast, the categories “Clinical pregnancy” and “Miscarriage” are based exclusively on cycles with a confirmed clinical pregnancy and reflect subsequent outcomes (i.e., ongoing pregnancy vs. loss).

**Table 3:** Associations between sperm DNA fragmentation, semen quality, embryological development, and clinical outcomes.

Parameter	SDF > 20% (*n* = 206)	SDF ≤ 20% (*n* = 664)	*p*-Value (Mann–Whitney U/χ^2^)	Correlation with SDF (Spearman’s r)	*p*-Value (Spearman)
Male age [years]	39.80 ± 6.60	37.60 ± 5.54	<0.001	0.162	<0.001
Male BMI [kg/m^2^]	26.8 ± 4.71	26.55 ± 4.32	0.755	−0.043	0.287
Sperm concentration [×10^6^/mL]	46.71 ± 62.59	53.93 ± 54.46	<0.001	−0.179	<0.001
Fast progressive motility [%]	8.91 ± 9.06	12.71 ± 9.73	<0.001	−0.271	<0.001
Slow progressive motility [%]	14.90 ± 10.94	21.47 ± 11.70	<0.001	−0.332	<0.001
Immotile sperm cells [%]	15.02 ± 6.75	18.01 ± 7.04	<0.001	0.398	<0.001
Normal sperm morphology [%]	2.00 ± 1.92	2.00 ± 1.88	0.083	−0.137	<0.001
Teratozoospermia Index (TZI)	2.84 ± 1.73	2.56 ± 1.68	0.020	–	–
Fertilization rate [%]	74.48 ± 26.72	79.42 ± 23.57	0.019	−0.084	0.012
Top-quality embryos (day 3) [%]	16.67 ± 25.51	19.09 ± 23.38	0.187	−0.069	0.040
Top-quality blastocysts [%]	21.30 ± 23.08	27.83 ± 26.12	<0.001	−0.110	0.001
Clinical pregnancy rate [%]	51.85% (84/162)	58.86% (322/547)	0.113	–	–

Footnotes: All values are presented as mean ± standard deviation (SD) unless otherwise indicated. Statistical significance was assessed using the Mann–Whitney U test for continuous variables and the χ^2^ test for clinical pregnancy rate; Spearman’s rank correlation was used to assess associations between continuous variables and SDF levels. *p*-values < 0.05 were considered statistically significant. Abbreviations: SDF—sperm DNA fragmentation; BMI—body mass index; TZI—teratozoospermia index; TQ—top-quality embryos or blastocysts; SD—standard deviation. Top-quality embryo (day 3) and blastocyst (day 5) rates refer to the proportion of embryos meeting morphological criteria for high quality. Clinical pregnancy was defined as the presence of a gestational sac with fetal heartbeat on ultrasound. The clinical pregnancy rate is based on all known historical cases of pregnancy. In contrast, distinctions between clinical pregnancy continuation and miscarriage (analyzed in separate sections) are based on confirmed pregnancies with follow-up data.

**Table 4:** Logistic regression analysis evaluating the association between sperm DNA fragmentation and reproductive outcomes in ICSI cycles.

Parameter	OR	95% CI	Coefficient (β)	Std. Error	Wald χ^2^	*p*-Value
Fertilization rate > 80%	0.984	0.971–0.997	−0.0162	0.0067	5.92	0.015
Top-quality embryos (Day 3)	0.983	0.966–1.001	−0.0169	0.0092	3.33	0.068
Top-quality blastocysts (Day 5)	0.975	0.958–0.992	−0.0255	0.0088	8.35	0.003
Clinical pregnancy (*n* = 377/659)	0.989	0.974–1.004	−0.0109	0.0077	2.02	0.154
Miscarriage (*n* = 49/377)	0.961	0.924–1.000	−0.0396	0.0204	3.76	0.052

Abbreviations: OR—odds ratio; CI—confidence interval; Std. Error—standard error; TQ3D—top-quality embryos on day 3; TQ5D—top-quality blastocysts on day 5. Note: Odds ratios reflect the effect of a 1% increase in sperm DNA fragmentation. Values < 1 indicate reduced odds of the outcome with increasing SDF. The clinical pregnancy rate was calculated for 659 transfers with known fetal heartbeat outcomes detected by ultrasound. Miscarriages are based on confirmed pregnancies with follow-up data.

## Data Availability

Source files for statistical analysis available from the corresponding author.

## References

[1] Agarwal A., Majzoub A., Baskaran S., Selvam M.K.P., Cho C.L., Henkel R., Finelli R., Leisegang K., Sengupta P., Barbarosie C. (2020). Sperm DNA Fragmentation: A New Guideline for Clinicians. World J. Men’s Health.

[2] Khalafalla K., Majzoub A., Elbardisi H., Bhathella A., Chaudhari A., Agarwal A., Henkel R., AlMarzooki T., Burjaq H., Arafa M. (2021). The Effect of Sperm DNA Fragmentation on Intracytoplasmic Sperm Injection Outcome. Andrologia.

[3] Majzoub A., Agarwal A., Esteves S.C. (2019). Clinical Utility of Sperm DNA Damage in Male Infertility. Panminerva Med..

[4] Esteves S.C., Zini A., Coward R.M., Evenson D.P., Gosálvez J., Lewis S.E.M., Sharma R., Humaidan P. (2021). Sperm DNA Fragmentation Testing: Summary Evidence and Clinical Practice Recommendations. Andrologia.

[5] Calogero A.E., Cannarella R., Agarwal A., Hamoda T.A.A. (2023). The Renaissance of Male Infertility Management in the Golden Age of Andrology. World J. Men’s Health.

[6] Casasus P., Luongo F.P., Haxhiu A., Orini M., Scupoli G., Governini L., Piomboni P., Buratini J., Canto M.D., Luddi A. (2024). Paternal Age Amplifies Cryopreservation-Induced Stress in Human Spermatozoa. Cells.

[7] Gisbert Iranzo A., Cano-Extremera M., Hervás I., Falquet Guillem M., Gil Juliá M., Navarro-Gomezlechon A., Pacheco-Rendón R.M., Garrido N. (2025). Sperm Selection Using Microfluidic Techniques Significantly Decreases Sperm DNA Fragmentation (SDF), Enhancing Reproductive Outcomes: A Systematic Review and Meta-Analysis. Biology.

[8] Luongo F.P., Perez Casasus S., Haxhiu A., Barbarulo F., Scarcella M., Governini L., Piomboni P., Scarica C., Luddi A. (2023). Exposure to Cumulus Cell Secretome Improves Sperm Function: New Perspectives for Sperm Selection In Vitro. Cells.

[9] Gill K., Machalowski T., Harasny P., Kups M., Grabowska M., Duchnik E., Sipak O., Fraczek M., Kurpisz M., Kurzawa R. (2022). Male Infertility Coexists with Decreased Sperm Genomic Integrity and Oxidative Stress in Semen Irrespective of Leukocytospermia. Antioxidants.

[10] Aitken R.J., Lewis S.E.M. (2023). REVIEW ARTICLE DNA Damage in Testicular Germ Cells and Spermatozoa. When and How Is It Induced? How Should We Measure It? What Does It Mean?. Andrology.

[11] Fernández J.L., Muriel L., Goyanes V., Segrelles E., Gosálvez J., Enciso M., LaFromboise M., De Jonge C. (2005). Simple Determination of Human Sperm DNA Fragmentation with an Improved Sperm Chromatin Dispersion Test. Fertil. Steril..

[12] Zini A., Sigman M. (2009). Are Tests of Sperm DNA Damage Clinically Useful?. Pros. and Cons. J. Androl..

[13] Gill K., Rosiak A., Gaczarzewicz D., Jakubik J., Kurzawa R., Kazienko A., Rymaszewska A., Laszczynska M., Grochans E., Piasecka M. (2018). The Effect of Human Sperm Chromatin Maturity on ICSI Outcomes. Hum. Cell.

[14] Gill K., Jakubik J., Rosiak-Gill A., Kups M., Lukaszuk M., Kurpisz M., Fraczek M., Piasecka M. (2019). Utility and Predictive Value of Human Standard Semen Parameters and Sperm Dna Dispersion for Fertility Potential. Int. J. Environ. Res. Public Health.

[15] Ribas-Maynou J., Novo S., Torres M., Salas-Huetos A., Rovira S., Antich M., Yeste M. (2022). Sperm DNA Integrity Does Play a Crucial Role for Embryo Development after ICSI, Notably When Good-Quality Oocytes from Young Donors Are Used. Biol. Res..

[16] Cissen M., Van Wely M., Scholten I., Mansell S., De Bruin J.P., Mol B.W., Braat D., Repping S., Hamer G. (2016). Measuring Sperm DNA Fragmentation and Clinical Outcomes of Medically Assisted Reproduction: A Systematic Review and Meta Analysis. PLoS ONE.

[17] Liu X., Zhao L., Lin Y., Liu Y. (2025). The Effect of Sperm DNA Fragmentation on Clinical Pregnancy and Miscarriage Following Intrauterine Insemination: Updated Systematic Review and Meta-Analysis. Urology.

[18] Panner Selvam M.K., Ambar R.F., Agarwal A., Henkel R. (2021). Etiologies of Sperm DNA Damage and Its Impact on Male Infertility. Andrologia.

[19] Farkouh A., Salvio G., Kuroda S., Saleh R., Vogiatzi P. (2022). Sperm DNA Integrity and Male Infertility: A Narrative Review and Guide for the Reproductive Physicians. Transl. Androl. Urol..

[20] Agarwal A., Farkouh A., Parekh N., Zini A., Arafa M. (2022). Sperm DNA Fragmentation: A Critical Assessment of Clinical Practice Guidelines. World J. Men’s Health.

[21] Al Omrani B., Al Eisa N., Javed M., Al Ghedan M., Al Matrafi H., Al Sufyan H. (2018). Associations of Sperm DNA Fragmentation with Lifestyle Factors and Semen Parameters of Saudi Men and Its Impact on ICSI Outcome. Reprod. Biol. Endocrinol..

[22] Osman A., Alsomait H., Seshadri S., El-Toukhy T., Khalaf Y. (2015). The Effect of Sperm DNA Fragmentation on Live Birth Rate after IVF or ICSI: A Systematic Review and Meta-Analysis. Reprod. Biomed. Online.

[23] Choi H.Y., Kim S.K., Kim S.H., Choi Y.M., Jee B.C. (2017). Impact of Sperm DNA Fragmentation on Clinical in Vitro Fertilization Outcomes. Clin. Exp. Reprod. Med..

[24] Bounartzi T., Dafopoulos K., Anifandis G., Messini C.I., Koutsonikou C., Kouris S., Satra M., Sotiriou S., Vamvakopoulos N., Messinis I.E. (2016). Pregnancy Prediction by Free Sperm DNA and Sperm DNA Fragmentation in Semen Specimens of IVF/ICSI-ET Patients. Hum. Fertil..

[25] Green K.A., Patounakis G., Dougherty M.P., Werner M.D., Scott R.T., Franasiak J.M. (2020). Sperm DNA Fragmentation on the Day of Fertilization Is Not Associated with Embryologic or Clinical Outcomes after IVF/ICSI. J. Assist. Reprod. Genet..

[26] Zhang F., Li J., Liang Z., Wu J., Li L., Chen C., Jin F., Tian Y. (2021). Sperm DNA Fragmentation and Male Fertility: A Retrospective Study of 5114 Men Attending a Reproductive Center. J. Assist. Reprod. Genet..

[27] Henkel R., Morris A., Vogiatzi P., Saleh R., Sallam H., Boitrelle F., Garrido N., Arafa M., Gül M., Rambhatla A. (2022). Predictive Value of Seminal Oxidation-Reduction Potential Analysis for Reproductive Outcomes of ICSI. Reprod. Biomed. Online.

[28] Ribeiro S., Sousa M. (2023). In Vitro Fertilisation and Intracytoplasmic Sperm Injection Predictive Factors: A Review of the Effect of Female Age, Ovarian Reserve, Male Age, and Male Factor on IVF/ICSI Treatment Outcomes. J. Bras. Reprod. Assist..

[29] Kaiyal R.S., Karna K.K., Kuroda S., Sgayer I., Shlush E., Vij S.C., Lundy S.D., Cannarella R. (2024). Sperm Chromatin Dispersion Assay Reliability and Assisted Reproductive Technology Outcomes: Systematic Review and Meta-Analysis. Andrology.

[30] Stringer J.M., Winship A., Liew S.H., Hutt K. (2018). The Capacity of Oocytes for DNA Repair. Cell. Mol. Life Sci..

[31] Sakkas D., Alvarez J.G. (2010). Sperm DNA Fragmentation: Mechanisms of Origin, Impact on Reproductive Outcome, and Analysis. Fertil. Steril..

[32] Ribas-Maynou J., Yeste M., Becerra-Tomás N., Aston K.I., James E.R., Salas-Huetos A. (2021). Clinical Implications of Sperm DNA Damage in IVF and ICSI: Updated Systematic Review and Meta-Analysis. Biol. Rev..

[33] Sedó C.A., Bilinski M., Lorenzi D., Uriondo H., Noblía F., Longobucco V., Lagar E.V., Nodar F. (2017). Effect of Sperm DNA Fragmentation on Embryo Development: Clinical and Biological Aspects. J. Bras. Reprod. Assist..

[34] El-Ela A.A., Kandeel A., Hassan E., Nasr M., Esam Y. (2022). Utility of Magnetic Activated Cell Sorting (MACS) in Assisted Reproduction. Al-Azhar Int. Med. J..

[35] Wang Q., Gu X., Chen Y., Wang X., Lv J., Yu M. (2023). The Effect of Sperm DNA Fragmentation on in Vitro Fertilization Outcomes of Unexplained Infertility. Clinics.

[36] Ni W., Xiao S., Qiu X., Jin J., Pan C., Li Y., Fei Q., Yang X., Zhang L., Huang X. (2014). Effect of Sperm DNA Fragmentation on Clinical Outcome of Frozen-Thawed Embryo Transfer and on Blastocyst Formation. PLoS ONE.

[37] Ješeta M., MyškovÃi M., ÁkovÃi J.Z., Crha I., Crha K., Chmelikova E., Kistanova E., Ventruba P. (2020). Can Oocytes Repair Fragmented DNA of Spermatozoa?. Med. J. Cell Biol..

[38] Meseguer M., Santiso R., Garrido N., García-Herrero S., Remohí J., Fernandez J.L. (2011). Effect of Sperm DNA Fragmentation on Pregnancy Outcome Depends on Oocyte Quality. Fertil. Steril..

[39] Ruiz-Díaz S., Mazzarella R., Navarrete-López P., Fernández-González R., de Frutos C., Maroto M., Cucala C., Beltrán-Breña P., Lombó M., Rizos D. (2023). Bull Spermatozoa Selected by Thermotaxis Exhibit High DNA Integrity, Specific Head Morphometry, and Improve ICSI Outcome. J. Anim. Sci. Biotechnol..

[40] Dehghanpour F., Khalili M.A., Mangoli E., Talebi A.R., Anbari F., Shamsi F., Woodward B., Doostabadi M.R. (2022). Free Centrifuge Sorting Method for High-Count Sperm Preparation Improves Biological Characteristics of Human Spermatozoa and Clinical Outcome: A Sibling Oocytes Study. Andrologia.

[41] Sharma S., Kabir M.A., Asghar W. (2022). Selection of Healthy Sperm Based on Positive Rheotaxis Using a Microfluidic Device. Analyst.

[42] Doostabadi M.R., Mangoli E., Marvast L.D., Dehghanpour F., Maleki B., Torkashvand H., Talebi A.R. (2022). Microfluidic Devices Employing Chemo- and Thermotaxis for Sperm Selection Can Improve Sperm Parameters and Function in Patients with High DNA Fragmentation. Andrologia.

[43] Ali A.H., Ajina T., Ben Ali M., Mehdi M. (2022). Efficacy of Density Gradient Centrifugation Technique (DGC) in Enhancing Sperm Cell DNA Quality for Assisted Reproductive Technique. Middle East Fertil. Soc. J..

[44] Sarbandi I.R., Lesani A., Moghimi Zand M., Nosrati R. (2021). Rheotaxis-Based Sperm Separation Using a Biomimicry Microfluidic Device. Sci. Rep..

[45] Vasilescu S.A., Ding L., Parast F.Y., Nosrati R., Warkiani M.E. (2023). Sperm Quality Metrics Were Improved by a Biomimetic Microfluidic Selection Platform Compared to Swim-up Methods. Microsystems Nanoeng..

[46] Ahmadi H., Aghebati-Maleki L., Rashidiani S., Csabai T., Nnaemeka O.B., Szekeres-Bartho J. (2023). Long-Term Effects of ART on the Health of the Offspring. Int. J. Mol. Sci..

[47] Santi D., Spaggiari G., Simoni M. (2018). Sperm DNA Fragmentation Index as a Promising Predictive Tool for Male Infertility Diagnosis and Treatment Management—Meta-Analyses. Reprod. Biomed. Online.

[48] Datta A.K., Campbell S., Diaz-Fernandez R., Nargund G. (2024). Livebirth Rates Are Influenced by an Interaction between Male and Female Partners’ Age: Analysis of 59 951 Fresh IVF/ICSI Cycles with and without Male Infertility. Hum. Reprod..

[49] Vaegter K.K., Lakic T.G., Olovsson M., Berglund L., Brodin T., Holte J. (2017). Which Factors Are Most Predictive for Live Birth after in Vitro Fertilization and Intracytoplasmic Sperm Injection (IVF/ICSI) Treatments? Analysis of 100 Prospectively Recorded Variables in 8,400 IVF/ICSI Single-Embryo Transfers. Fertil. Steril..

[50] Repalle D., Saritha K.V., Bhandari S., Chittora M., Choudhary J. (2022). Role of Female Age in Regulating the Effect of Sperm DNA Fragmentation on the Live Birth Rates in Intracytoplasmic Sperm Injection Cycles with Own and Donor Oocytes. J. Hum. Reprod. Sci..

[51] World Health Organization (2021). WHO Laboratory Manual for the Examination and Processing of Human Semen.

[52] Stavros S., Potiris A., Molopodi E., Mavrogianni D., Zikopoulos A., Louis K., Karampitsakos T., Nazou E., Sioutis D., Christodoulaki C. (2024). Sperm DNA Fragmentation: Unraveling Its Imperative Impact on Male Infertility Based on Recent Evidence. Int. J. Mol. Sci..

[53] Palermo G. (1992). Pregnancies after Intracytoplasmic Injection of Single Spermatozoon into an Oocyte. Lancet.

[54] Tannus S., Son W.Y., Gilman A., Younes G., Shavit T., Dahan M.H. (2017). The Role of Intracytoplasmic Sperm Injection in Non-Male Factor Infertility in Advanced Maternal Age. Hum. Reprod..

[55] Amirjannati N., Mohazzab A., Fathalian M., Akhavizadegan H. (2024). Comparison of Embryological Results of Microinjection in Two Groups of Men with and without Requesting Sperm DNA Fragmentation Index Measurement. Bio. Med. Res. Int..

[56] Siddhartha N., Reddy N.S., Pandurangi M., Muthusamy T., Vembu R., Kasinathan K. (2019). The Effect of Sperm DNA Fragmentation Index on the Outcome of Intrauterine Insemination and Intracytoplasmic Sperm Injection. J. Hum. Reprod. Sci..

[57] Sun T.C., Zhang Y., Li H.T., Liu X.M., Yi D.X., Tian L., Liu Y.X. (2018). Sperm DNA Fragmentation Index, as Measured by Sperm Chromatin Dispersion, Might Not Predict Assisted Reproductive Outcome. Taiwan. J. Obstet. Gynecol..

